# Benign Giant Cell Lesion of C1 Lateral Mass: A Case Report and Literature Review

**DOI:** 10.3390/brainsci9050105

**Published:** 2019-05-08

**Authors:** Christopher Heinrich, Vadim Gospodarev, Albert Kheradpour, Craig Zuppan, Clifford C. Douglas, Tanya Minasian

**Affiliations:** 1 Loma Linda University School of Medicine, Loma Linda, CA 92354, USA; ckheinrich@llu.edu; 2 Center for Perinatal Biology, Department of Basic Sciences, Loma Linda University, 11234 Anderson Street, Room 2567, Loma Linda, CA 92354, USA; 3 Department of Pediatric Hematology/Oncology, Loma Linda University Medical Center, 11234 Anderson Street, Loma Linda, CA 92354, USA; AKheradp@llu.edu; 4 Department of Pathology, Loma Linda University Medical Center, 11234 Anderson Street, Loma Linda, CA 92354, USA; CZuppan@llu.edu; 5 Department of Neurosurgery, Loma Linda University Medical Center, 11234 Anderson Street, Room 2556, Loma Linda, CA 92354, USA; CCDougla@llu.edu (C.C.D.); TMinasian@llu.edu (T.M.)

**Keywords:** pain, spine, spinal disease, transoral, atlantoaxial, surgery

## Abstract

Primary osseous tumors of the spinal column account for approximately 1% of the total number of spinal tumors found in the pediatric patient population. The authors present a case of a C1 benign giant cell lesion that was incidentally found in a 15-year-old patient. A transoral biopsy was performed followed by treatment with denosumab, with definitive management in the form of transoral tumor resection with subsequent occiput-cervical three posterior instrumented fusion. The patient tolerated all of the procedures well, as there were no post-operative complications, discharged home neurologically intact and was eager to return to school when assessed during a follow-up visit in clinic. Osteolytic lesions affecting the cervical spine are rare in the pediatric population. It is of utmost importance to have sufficient background knowledge in order to formulate a differential diagnosis, as well as an understanding of principles underlying surgical techniques required to prevent occipital-cervical instability in this patient population. The information presented will guide surgical decision-making by identifying the patient population that would benefit from neurosurgical interventions to stabilize the atlantoaxial junction, in the context of rare osteolytic conditions affecting the cervical spine.

## 1. Introduction

Spinal tumors comprise approximately 5–10% of all pediatric central nervous system (CNS) tumors [1,2,3]. Primary osseous tumors of the spinal column only account for approximately 1% of the total number of spinal tumors [4]. The authors present a case of a C1 benign giant cell lesion, that was incidentally found in a 15-year-old patient. Despite the benign pathologic diagnosis, the unusual location and behavior of the lesion warranted an interesting surgical approach. Including a transoral biopsy first, followed by treatment with Denosumab, and finally definitive management in the form of transoral tumor resection with subsequent occiput-cervical three posterior instrumented fusion. Due to the complex anatomy of the atlantooccipital and atlantoaxial joint spaces and related structures, treatment of a mass in this region, whether benign or malignant, would necessitate non-surgical management as first-line treatment. However, if a mass is deemed to be locally destructive, resistant to medical management, or both, then a definitive surgical approach is necessary. Regardless of treatment paradigm, effective dissolution of a lytic lesion in this region can destabilize the atlantooccipital and atlantoaxial joints endangering key neurovascular structures. Because of this, stabilization of these joints using instrumented fusion is key to prevention of patient morbidity and potential mortality. 

## 2. Case Description

**Figure 1:**
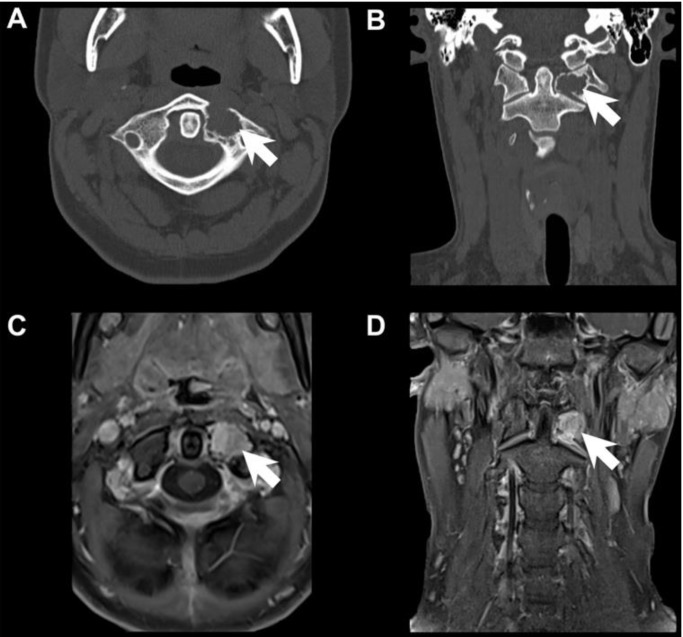
Pre-Operative Imaging Studies. CT of cervical spine, axial view, demonstrating the radiolucent, ovoid-shaped, lytic lesion (white arrow) arising in the left lateral mass of C1 between the anterior tubercle and the transverse process (**A**). CT of cervical spine, coronal view, demonstrating the lesion (white arrow) (**B**). Post-contrast MRI T1, axial view, demonstrating an enhancing osseous lesion (white arrow) at the left lateral mass of C1 with cortical breach and extension into the left lateral atlantodental joint space (**C**). Post-contrast MRI T1, coronal view, of the lesion (white arrow) (**D**).

A 15-year-old female with no significant past medical history presented after being struck in the face by a ball while playing water polo. The patient felt pain in her jaw, which was the chief complaint when she presented to the emergency department. Upon neurological assessment, the patient complained of midline tenderness from the skull base to midline cervical spine over C3; denied headaches, changes in vision, speech or swallowing, extremity weakness or paresthesias. A maxillofacial computed tomography (CT) scan did not show evidence of an acute facial fracture. However, the CT scan did reveal a radiolucent, ovoid-shaped lytic lesion arising in the left lateral mass of C1, between the anterior tubercle and the transverse process. Magnetic resonance imaging (MRI) studies further confirmed an enhancing osseous lesion at the left lateral mass of C1, with cortical breach and extension into the left lateral atlantodental joint space (Figure 1). Of note, three years prior, patient had a CT cervical spine which, upon retrospective review, demonstrated a similar but much smaller lesion.

Differential diagnoses underlying this vertebral cortical erosion included those of infectious etiology, as well as oncologic lesions, such as giant cell tumor of bone, aneurysmal bone cyst, osteoblastoma, osteosarcoma or even Langerhans histiocytosis (LCH). Oncology recommended that the cervical spine lesion be biopsied for tissue diagnosis. Due to the unusual location of the lesion and risk of locally aggressive pathology, or possible tumor seeding along the biopsy track, interventional radiology was unable to perform a CT guided needle biopsy. It was therefore decided that the patient would require open neurosurgical biopsy for diagnosis. 

Due to the anterior and lateral location of the vertebral lesion, an anterior transoral approach to the C1 lesion was performed, in order to obtain a sufficient amount of the contrast enhancing component of the mass for pathologic diagnosis. The transoral approach was performed in a multidisciplinary fashion, during which the otolaryngology team used direct visualization, as well as stereotactic navigation, to expose the C1 anterior tubercle on the left side. Once exposure was completed, neurosurgery team utilized a matchstick burr to then drill the anterior outer cortex of C1. Multiple specimens from the fibrous tumor were taken, with curettes and pituitary forceps. 

**Figure 2:**
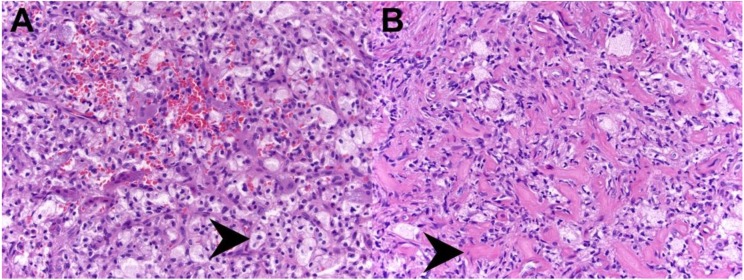
Histopathology. Hematoxylin and eosin (H&E) stained tissue from the initial biopsy which showed moderate prominence of histiocytic foam cells (black arrowhead), intermixed spindle cells, and scattered giant cells (**A**). H&E stained tissue following Denosumab therapy, showing disappearance of the giant cells, few foamy histiocytes, and predominance of short spindled cells in a collagenous stroma (black arrowhead) (**B**).

The sampled tissue did not show features of osteoblastoma or osteosarcoma, nor were there features of LCH or signs of infection. In the sampled region, the lesion consisted of a proliferation of nondescript stromal cells with intermixed multinucleated giant cells, and occasional clusters of foamy histiocytes (Figure 2). Special testing for giant cell tumor of bone (G34W staining) was negative, as was fluorescence in situ hybridization (FISH) testing for Ubiquitin Specific Peptidase 6 (USP6), making a primary form of aneurysmal bone cyst unlikely. However, due to the aggressive nature of the patient’s osteolytic lesion and the significant risk for atlantoaxial instability associated with its location, it was decided to start the patient on Denosumab. Denosumab is an osteoclast inhibiting pharmaceutical agent, which was administered to the patient in order to stabilize and consolidate the lesion. Samples of the patient’s lesion were also sent out to a nationally recognized expert bone pathologist, whose findings were most consistent with benign giant cell rich lesion with histiocytes. 

The patient was re-assessed three months postoperatively and MRI studies revealed that there was no interval decrease in the size of the tumor. In fact, there was a slight progression of the lesion anteriorly, despite treatment with Denosumab. After presenting the patient’s case at our institution’s multidisciplinary tumor board, it was decided to offer the patient a gross total resection of the offending lesion. This would inherently lead to significant atlantoaxial instability, therefore a posterior occiput to cervical three instrumented fusion was also warranted.

**Figure 3:**
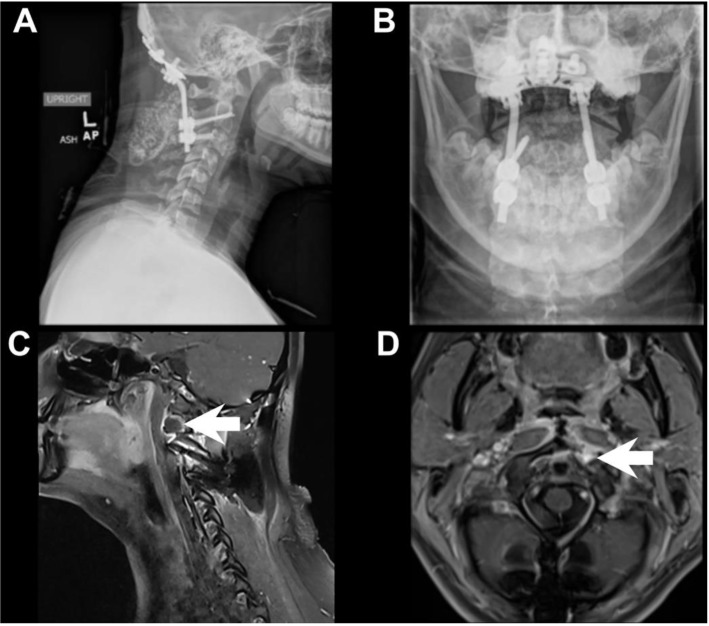
Post-Operative Imaging Studies. Lateral view X-ray of the cervical spine demonstrating hardware on the occiput connected by rods to pedicle screws in C2 and lateral mass screws in C3 bilaterally (**A**). Open mouth odontoid view X-ray demonstrates normal symmetry of C1 and C2 articulation (**B**). Post-contrast MRI T1, sagittal view, demonstrates minimal rim-enhancement within the left lateral C1 representing mild residual tumor (white arrow) (**C**). Post-contrast MRI T1, axial view, of the residual lesion (white arrow) (**D**).

The transoral approach was performed in a multidisciplinary fashion, during which the otolaryngology team used direct visualization as well as stereotactic navigation, to expose the cervical vertebrae through the posterior pharynx. Fibrous tumor was identified and dissected until superior, inferior, and lateral margins of tumor resection were confirmed grossly, with fluoroscopy, and neuronavigation. Additional C1 anterior tubercle eccentric towards the right side was also taken, to include a normal bony margin. A small rim of tumor adherent to the vertebral artery was left behind. After the otolaryngology team closed the posterior pharynx, the patient was carefully turned prone, maintaining spinal precautions. Base of the occiput to cervical three was then exposed. C2 pedicle screws were placed. C3 lateral mass screws were placed. An occipital plate was sized. Screws into the occiput were placed. Fluoroscopy confirmed excellent position and spinal alignment. There were no post-operative complications and the patient was discharged home in good condition. Pathologic examination of the resected material at this time showed complete disappearance of the giant cells, due to Denosumab therapy, with the remaining lesional tissue resembling benign fibrous histiocytoma (Figure 2). Post-operative imaging studies revealed a stable posterior cervical spine construct, along with minimal rim-enhancement along the vertebral artery, as expected (Figure 3). At a three-week follow up visit in clinic, the patient’s incisions were healing well, she was neurologically intact, tolerating regular diet, and was eager to return to school.

## 3. Discussion

The differential diagnosis for a spinal column tumor in a pediatric patient is broad, ranging from primary osseous lesions like osteochondromas and osteosarcomas, to tumors of soft tissue origin like rhabdomyosarcoma or neuroblastoma. If the patient’s age is taken into consideration, the most common primary osseous tumors in the pediatric spinal column are as follows: 0–5 years of age: eosinophilic granuloma; 5–10 years of age: aneurysmal bone cyst, eosinophilic granuloma, osteoblastoma, osteoid osteoma, osteosarcoma, and Ewing sarcoma; lastly 10–20 years of age: aneurysmal bone cyst, osteochondroma, osteoid osteoma, osteosarcoma, Ewing sarcoma [5]. 

The most common primary osseous tumor in children is osteochondroma, which makes up about 4% of solitary spinal column tumors. They are benign lesions that consist of an abnormal outgrowth of bone with cartilaginous cap [4]. When these occur in the spine, they occur most often in the cervical spine and must be considered when evaluating a solitary spinal mass in a pediatric patient [6,7,8]. Of note, about 10% of these tumors will undergo malignant transformation to osteosarcoma. These tumors are usually seen in patients from ages 10–20 and make up about 5% of all spinal lesions [4,9]. 

The most common malignant primary bone tumor seen in the spinal column of pediatric patients is Ewing sarcoma. Interestingly, these lesions can either arise as primary spinal lesions, or metastasize from other locations to the spine, a characteristic not usually seen in other primary osseous lesions. Ewing sarcoma has a predilection for the sacrum and are found less commonly in the lumbar, thoracic spine, and cervical spine, with decreasing frequency [10]. Osteoblastoma and osteoid osteoma are very pathologically similar primary osseous lesions. They differ mainly in size and behavior, and spinal lesions are most commonly found in the lumbar region [11]. Osteoblastomas are larger, generally greater than 2 cm, and tend to be locally aggressive whereas osteoid osteomas tend to be smaller than 1 cm and more latent, tending to “burn out” over time [12,13].

Another benign primary osseous lesion that can arise in the spine is the aneurysmal bone cyst. These lesions typically present in patients that are 20 years of age or younger and usually affect the lumbar spine [14,15]. Of note, these lesions can invade the pedicles and vertebral bodies on multiple vertebral levels [16]. Lastly, Langerhans cell histiocytosis is a common finding that can be seen in any bone, but occurs in the spine in approximately 10%–15% of cases [5]. The thoracic and cervical spinal regions tend to be the most common locations for this entity [17,18].

Management of most osseous spinal lesions involves en bloc resection with clear margins to avoid tumor recurrence and can be followed by adjuvant therapy, if indicated by pathologic analysis [19,20]. However, careful consideration must be taken to determine the best approach to a spinal column tumor, especially if it is in the cervical region. Tumors in the posterior aspect of the cervical spine usually require a posterior approach with the patient in the prone position [21]. Tumors along the anterior aspect of the spine present a more difficult management dilemma. The anatomy in this region is highly complex and involves many critical neurovascular structures that must be preserved to maintain the patient’s post-operative functional status. The transoral approach, which was originally pioneered by Kanavel in 1917, allows for midline access to a wider surgical field with access to multiple cervical vertebrae from C1 to C4 with less compromise to key neurovascular structures [22,23]. This approach has been used in the past to access the anterior cervical spine as noted by Tuite et al. in their report on 27 pediatric patients with occipitocervical junction pathology [24]. They also note that while this approach could have associated morbidity, such as temporomandibular joint dislocation, pharyngeal infection, and swallowing complications, when a standard transoral approach is completed and the hard palate is not compromised, the approach is safe and well tolerated [24,25]. Interestingly, this anatomic region has also been approached via the trans-nasal route as noted by Grammatica et al. This approach is well suited for patients with smaller oral cavities, that would limit the transoral approach and may represent decreased patient morbidity due to the lack of oral retraction during the procedure [26]. However, in cases where tumor pathology is quite lateral in its extent, trans-nasal approach would not allow for proper visualization of key neurovascular structures and would hinder the gross total resection. 

To prevent occipitocervical instability, it is often necessary to reconstruct elements of the cervical vertebral column after tumor extirpation. Instability is usually due to a combination of destruction of local anatomy by the tumor and correlates to extent of bony and ligamentous removal during surgery [21]. In these cases, posterior cervical fusion is warranted. In the patient presented here, given extent of bony C1 removal, compromising the ability to place hardware into C1, an occiput to cervical three construct was required for adequate stabilization.

## 4. Conclusions

Here we report a case of an osteolytic lesion that was incidentally found in the cervical spine of a pediatric patient. The relevant differential diagnosis, as well as surgical techniques involved in treating this patient, have been thoroughly discussed. Anatomy of the atlantooccipital and atlantoaxial joint spaces is complex and medical management of osteolytic lesions discovered in this area should be considered as first-line treatment. However, when the offending lesion is locally aggressive and does not respond to medical management, one must consider surgical intervention for attempted gross total resection, with stabilization of the cranio-vertebral junction. Stabilization of the aforementioned junction using instrumented fusion is key to prevention of patient morbidity and mortality. It is our hope that the information presented will guide surgical decision-making. It is critical to be able to correctly identify the patient population that would most likely benefit from neurosurgical interventions, in the context of rare osteolytic conditions affecting the cervical spine.
